# Population attributable fractions for Type 2 diabetes: an examination of multiple risk factors including symptoms of depression and anxiety

**DOI:** 10.1186/s13098-018-0387-5

**Published:** 2018-11-22

**Authors:** Kiyuri Naicker, Douglas Manuel, Simon Øverland, Jens C. Skogen, Jeffrey A. Johnson, Børge Sivertsen, Ian Colman

**Affiliations:** 1 School of Epidemiology and Public Health, University of Ottawa, 600 Peter Morand Cres., Room 308C, Ottawa, ON K1G 5Z3 Canada; 2 Ottawa Hospital Research Institute, Ottawa, ON Canada; 3 Department of Health Promotion, Norwegian Institute of Public Health, Bergen, Norway; 4 Department of Psychosocial Science, University of Bergen, Bergen, Norway; 5 Center for Alcohol and Drug Research, Stavanger University Hospital, Stavanger, Norway; 6 School of Public Health, University of Alberta, Edmonton, AB Canada; 7 Department Research and Innovation, Helse Fonna HF, Haugesund, Norway; 8 The Regional Centre for Child and Youth Mental Health and Child Welfare, Uni Research Health, Bergen, Norway

**Keywords:** Type 2 diabetes, Attributable fractions, Depression, Anxiety, Metabolic risk

## Abstract

**Background:**

Population attributable fractions (PAFs) are frequently used to quantify the proportion of Type 2 diabetes cases due to single risk factors, an approach which may result in an overestimation of their individual contributions. This study aimed to examine Type 2 diabetes incidence associated with multiple risk factor combinations, including the metabolic syndrome, behavioural factors, and specifically, depression and anxiety.

**Methods:**

Using data from the population-based HUNT cohort, we examined incident diabetes in 36,161 Norwegian adults from 1995 to 2008. PAFs were calculated using Miettinen’s case-based formula, using relative risks estimated from multivariate regression models.

**Results:**

Overall, the studied risk factors accounted for 50.5% of new diabetes cases (78.2% in men and 47.0% in women). Individuals exposed to both behavioural and metabolic factors were at highest risk of diabetes onset (PAF = 22.9%). Baseline anxiety and depression contributed a further 13.6% of new cases to this combination. Men appeared to be particularly vulnerable to the interaction between metabolic, behavioural and psychological risk factors.

**Conclusion:**

This study highlights the importance of risk factor clustering in diabetes onset, and is the first that we know of to quantify the excess fraction of incident diabetes associated with psychological risk factor interactions.

**Electronic supplementary material:**

The online version of this article (10.1186/s13098-018-0387-5) contains supplementary material, which is available to authorized users.

## Background

**Fig. 1:**
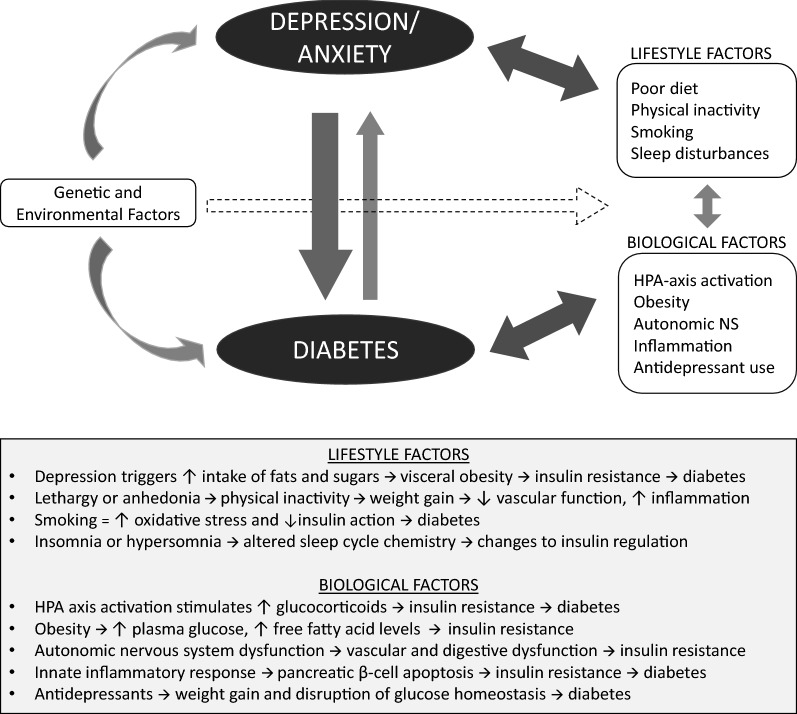
Pathological mechanisms linking depression and anxiety to diabetes outcomes through lifestyle and biological risk factors

Over 400 million adults worldwide are currently diagnosed with Type 2 diabetes, a prevalence that has more than doubled over the past three decades and continues to rise [1]. To address what is increasingly referred to as a global health crisis, many countries are investing in programs aimed at reducing the main modifiable risk factors of diabetes: obesity, smoking, unhealthy eating, and physical inactivity. Alongside these traditional risk factors, psychological factors are increasingly noted as being important to the pathogenesis of diabetes [2]. It has long been suggested that the risk of Type 2 diabetes is elevated by depression [3], and a growing evidence base has established depression as an independent risk factor for diabetes onset. Depression may trigger diabetogenic processes by influencing both behavioural and biological pathways (Fig. 1). This body of research extends to the effects of depression on obesity [4], physical activity [5], insulin resistance [6], and glycemic control [7]. Anxiety disorders represent a less frequently studied risk factor for diabetes onset, but anxiety is shown to elicit similar effects on the dysregulation of metabolic and inflammatory processes as depression (e.g., through HPA-axis activation [8] or cytokine-mediated autoimmune responses [9]) (Fig. 1). While major depression and generalized anxiety disorder share four common symptoms (according to DSM-V criteria) [10], they are also characterized by heterogenous symptoms that may act in direct opposition to one other (e.g., insomnia versus hypersomnia, restlessness versus motor retardation). Anxiety is unique in that some of its symptoms may even confer protective benefits (e.g., anxiety has been associated with low blood pressure in a large community-based sample of individuals (N = 60,700) [11]). Notably, anxiety disorders and depression are seen to occur together in over 50% of primary care cases [12]. Modalities for the treatment of patients with depression comorbid with diabetes include psychosocial interventions (e.g., cognitive-behaviour therapy), pharmacological interventions (e.g., selective-serotonin reuptake inhibitors), and collaborative care models (e.g., stepped care or algorithm-based approaches), all of which have been demonstrated to achieve improvements over usual-care for diabetes on key outcomes [13, 14].

In 2008, Cosgrove et al. estimated the attributable risk of developing Type 2 diabetes following an episode of depression to be 20%, and the total population fraction of diabetes cases attributable to depression at 4% [15]. Population attributable fractions (PAFs) are a common and useful method of quantifying the burden of disease attributable to a specific risk factor, and take into account both the strength of the association between the risk factor and disease and the wider population health impact of that risk factor [16]. PAFs have been used to describe the proportion of Type 2 diabetes cases due to physical inactivity [16], obesity [17], sugar intake [18], metabolic factors [19], and specific genetic variants [20]. For example, a recent study concluded that the proportion of diabetes cases attributable to sugar-sweetened beverages was 8.7% (3.9% to 12.9%) in the United States [18]. The authors concluded that, under an assumption of causality, these beverages are expected to cause approximately 2 million excess diabetes cases over 10 years in the US [18].

Although researchers are often compelled to quantify the PAFs for individual factors, the reality is that risk factors often cluster and may interact to amplify the risk associated with any one factor. Various studies have documented that individuals with certain combinations of risk factors are at higher risk of cardiovascular disease [21–23], and that screening can be more efficient if multiple risk factors are considered [24]. As the bulk of chronic diseases and subsequent mortality are attributable to risk factor combinations, the calculation of the PAF for any risk factor individually may greatly overestimate the risk associated with that factor [25]. A relatively sparse body of research has used a multiple risk factor approach to quantify the burden of diabetes onset [26], and no studies to date appear to have included an examination of depression and anxiety. The purpose of this study was to determine the PAF for multiple risk factor combinations for Type 2 diabetes, including symptoms of depression and anxiety, in a population-based sample of Norwegian adults.

## Methods

### Data source and participants

This study used cohort data from The Norwegian Nord-Trøndelag Health Study (HUNT Study), a population-based survey conducted over 3 waves: HUNT 1 (1984–86), HUNT 2 (1995–97) and HUNT 3 (2006–08). The HUNT study was initially established to address four major health outcomes (i.e., diabetes, hypertension, tuberculosis, and quality of life). The scope has broadened over time to include prevalence and incidence of disease, health determinants, and a state-of-the-art biobank [27]. Every citizen of Nord-Trøndelag county aged 20+ years was invited to participate. A total of 77,212 (89.4%) of those invited participated in the first wave of the survey, followed by 65,237 (69.5%) in HUNT 2, and 50,807 in HUNT 3 (54.1%) [27]. Further details of the HUNT cohort profile and methodology are well-described elsewhere [27].

The longitudinal HUNT 2–3 cohort consisted of 37,071 individuals who participated in the two most recent waves of the study. Our study sample consisted of participants in this cohort who did not have a confirmed diagnosis of diabetes (Type 1, Type 2, or LADA) at baseline in 1995–97 (HUNT 2), totaling 36,161 individuals. All participants provided written informed consent, and study protocols were approved by Norway’s Regional Committee for Medical and Health Research Ethics (2014/2160/REK West).

### Primary outcome: Type 2 diabetes onset

Our primary outcome was new Type 2 diabetes cases occurring between 1995 and 2008. Diabetes incidence was assessed in 2006–08 (HUNT 3), and confirmed based on results of three repeated laboratory tests (fasting plasma glucose, oral glucose tolerance, and serum HbA1c levels). All individuals were additionally confirmed to be anti-glutamic acid decarboxylase and anti-insulinoma-associated negative (to exclude LADA cases).

### Risk factors for Type 2 diabetes onset

Our primary study risk factors were divided into three categories: metabolic, behavioural, and psychological. We classified the metabolic syndrome according to the International Diabetes Federation consensus worldwide definition [28]. Central obesity was indicated if waist circumference exceeded 94 cm for men or 79 cm for women. Low high-density lipoprotein (HDL) cholesterol was indicated at levels below 40 mg/dL (1.03 mmol/L) in men and 50 mg/dL (1.29 mmol/L) in women. Hypertension was indicated at a systolic blood pressure > 130 mmHg or diastolic > 85 mmHg. Random plasma glucose ≥ 200 mg/dL (11.1 mmol/L) was used to indicate hyperglycemia (as fasting glucose measurements were not available) [29]. The metabolic syndrome was defined as having central obesity plus any two of the latter four factors [28].

Two major behavioural factors in the onset of diabetes were included: physical inactivity and smoking. Individuals were classified as physically inactive if they fell below the cut-off of 150 min of moderate or 60 min of vigorous physical activity per week (corresponding to World Health Organization guidelines [30]). Smoking was included if either former or current daily smoking were reported, as both have known associations with diabetes onset [31], and included as total number of packyears smoked (calculations based on 20 cigarettes/pack). The presence of either of these two factors were considered an indicator of behavioural risk in calculations of population attributable fractions.

Symptoms of depression and anxiety were measured using a 7-item mental health index (CONOR-MHI). These items consisted of widely validated items from the General Health Questionnaire and the Hopkins Symptom Checklist (HSCL). The index was evaluated against both the HSCL-10 and the Hospital Anxiety and Depression Scale (HADS) within both the HUNT Study and Oslo Health Study cohorts, and found to have strong accuracy when tested against each of these scales as a gold standard (AUC = 0.902 and AUC = 0.909, respectively) [32]. Individuals were classified as having depression or anxiety if their mean scores on depression or anxiety items fell above the recommended cut-off of 2.15 [32].

### Additional covariates

Age was considered as a continuous covariate and specified using restricted cubic splines in multivariate regression analyses, to account for the non-linear relationship between age and diabetes onset (as per Harrell’s method [33]). Education was classified as having completed any post-secondary versus no higher education. The presence of any additional chronic conditions (i.e., asthma, angina pectoris, any type of cancer or thyroid disease) was included dichotomously.

### Statistical analysis

Relative risks were estimated using univariate and multivariate regression models. Analyses were performed using generalized linear mixed models for binary data in STATA 14.0. All models controlled for age and were stratified by sex. Adjusted models were constructed using all covariates and all possible two-way interaction terms. Models were built using backwards selection, and all variables significant at the α = 0.10 level were included in the final models. Regressions were conducted following imputation of missing covariate values, under the multiple imputation by chained equations (MICE) method [34]. This technique created 30 imputed datasets, from which reported estimates were computed. The amount of data missing was low (1%–7%) on almost all covariates.

The population attributable fraction (PAF) was then calculated for strata in which the relative risk was greater than 1.0 and significant at the α = 0.05 level. PAFs were calculated using Miettinen’s case-based formula

$${\text{PAR}}_{\text{i}} = \left( {{\text{RR}}_{\text{i}} - 1} \right)/{\text{RR}}_{\text{i}} \times {\text{ CF}}_{\text{i}} ,$$

where RR_i_ equals the relative risk associated with the strata in question and CF_i_ denotes the case fraction (CF = number of exposed cases/total number of cases) [35]. This approach was selected over the original formula for calculating attributable fractions popularized by Levin [36], as it produces internally valid estimates in the presence of confounding and when adjusted relative risks must be used. A sensitivity analysis comparing the two approaches was also conducted (see Additional file 3: Table S3).

## Results

Of the 36,161 participants, 55% were female and the majority were between 20 and 61 years of age (Table 1). Men and women differed to some extent with respect to all baseline diabetes risk factors, with the most pronounced differences corresponding to higher rates of central obesity and physical inactivity in women, and higher rates of hypertension and elevated triglycerides in men (Table 1). Overall, symptoms of either depression or anxiety were present in 8.8% of individuals at baseline. A total of 1324 incident cases of diabetes were reported during the study period, 655 cases occurring in women and 669 cases in men.

**Table 1:** Baseline characteristics of the HUNT 2–3 cohort

	Women (n = 20,014)	Men (n = 16,147)	All (N = 36,161)
Demographic factors			
Age (mean, SD)	46.8 (13.7)	47.3 (13.1)	47.0 (13.4)
20–34	22.1%	19.5%	20.9%
35–44	24.3%	24.2%	24.3%
45–54	26.7%	27.9%	26.7%
55–64	17.0%	17.7%	17.0%
≥ 65	11.5%	10.6%	11.1%
Education (any post-secondary)	33.2%	30.1%	31.8%
Metabolic factors			
Central obesity^a^	47.9%	36.9%	42.9%
Hypertension^b^	46.2%	66.5%	55.3%
Triglycerides (high)	28.5%	48.5%	37.4%
HDL cholesterol (low)	24.9%	29.1%	26.8%
Plasma glucose (high)	2.1%	3.3%	2.6%
Metabolic syndrome present	20.2%	23.1%	21.5%
Behavioural factors			
Smoking (packyears, mean)	12.8 (10.2)	17.2 (13.6)	14.9 (12.1)
Former smoking (daily)	24.2%	33.5%	28.4%
Current smoking (daily)	27.9%	24.5%	26.4%
Physical inactivity^c^	54.3%	48.2%	51.5%
Psychological factors			
Depression only	1.9%	1.3%	1.6%
Anxiety only	5.3%	4.5%	4.9%
Concurrent dep–anx	2.6%	1.9%	2.3%
All (dep or anx)	9.8%	7.7%	8.8%

^a^Waist circumference ≥ 94 cm for men; ≥ 80 for women

^b^Systolic blood pressure > 130 mmHg or diastolic > 85 mmHg

^c^Less than 150 min of moderate or 60 min of vigorous physical activity per week

In univariate analysis, the relative risk of diabetes incidence increased more than fivefold in the presence of the metabolic syndrome (Table 2). Smoking was associated with diabetes incidence in men only (RR = 1.63 [95% CI 1.33, 2.00]), as were depression and comorbid depression-anxiety (RR = 1.65 [95% CI 1.24, 2.20], and RR = 2.07 [95% CI 1.41, 3.05], respectively). Physical inactivity was associated with a 1.5-fold increase in diabetes risk for both sexes. Symptoms of anxiety alone were not associated with elevated diabetes risk in either sex. A dose–response increase in diabetes risk was also observed, with respect to the number of metabolic, behavioural and mental health risk factors present. This ranged from two factors (RR = 2.62 [95% CI 1.97, 3.50]) to ≥ 4 factors (RR = 11.89 [95% CI 9.22, 15.34]).

**Table 2:** Multivariate relative risk of incident Type 2 diabetes in HUNT 2–3 cohort, by sex

	Women (n = 20,014)		Men (n = 16,147)		All (n = 36,161)	
	RR	95% CI	RR	95% CI	RR	95% CI
Demographic factors						
Age						
20–34	Ref	Ref	Ref	Ref	Ref	Ref
35–44	2.42	1.57, 3.74	2.97	1.90, 4.63	2.68	1.97, 3.65
45–54	3.53	2.33, 5.36	4.45	2.90, 6.83	3.82	2.84, 5.14
55–64	4.23	2.76, 6.48	5.68	3.68, 8.77	4.72	3.50, 6.37
≥ 65	2.97	1.88, 4.70	5.41	3.43, 8.52	3.88	2.83, 5.32
Education (any post-secondary)	1.20	0.96, 1.50	1.19	1.00, 1.44	1.20	1.04, 1.38
Metabolic factors						
Central obesity^a^	4.77	3.64, 6.25	3.31	2.75, 3.99	3.59	3.08, 4.18
Hypertension^b^	1.67	1.37, 2.04	1.49	1.22, 1.81	1.54	1.34, 1.77
Triglycerides	1.98	1.63, 2.41	1.79	1.47, 2.19	1.77	1.55, 2.03
HDL cholesterol	2.13	1.78, 2.54	1.84	1.57, 2.16	1.86	1.66, 2.10
Plasma glucose	3.68	2.40, 4.03	5.58	3.31, 6.90	4.05	3.55, 4.62
Behavioural factors						
Smoking						
Never	Ref	Ref	Ref	Ref	Ref	Ref
Former (daily)	0.87	0.71, 1.07	1.43	1.18, 1.72	1.13	0.99, 1.29
Current (daily*)*	0.99	0.82, 1.22	1.50	1.22, 1.85	1.21	1.05, 1.39
Physical inactivity^c^	1.05	0.88, 1.24	1.38	1.18, 1.61	1.23	1.08, 1.36
Psychological factors						
Depression only	1.14	0.63, 2.08	1.94	1.34, 2.83	1.37	1.01, 1.87
Anxiety only	1.24	0.92, 1.68	1.16	0.62, 2.16	1.16	0.76, 1.80
Concurrent Dep–anx	0.93	0.56, 1.55	1.50	1.13, 2.00	1.36	1.10, 1.68

^a^Waist circumference ≥ 94 cm for men; ≥ 80 for women

^b^Systolic blood pressure > 130 mmHg or diastolic > 85 mmHg

^c^Less than 150 min of moderate or 60 min of vigorous physical activity per week

**Fig. 2:**
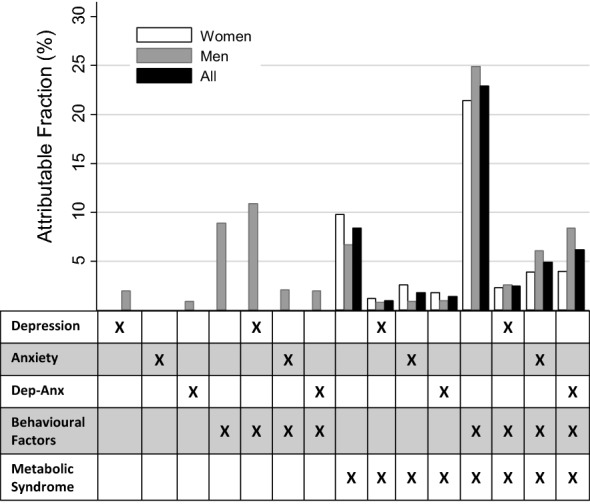
Attributable fractions for Type 2 diabetes onset in Norwegian adults associated with multiple risk factors (N = 36,161)

The final multivariate model included education and age as covariates, and excluded the presence of other chronic conditions. The presence of the metabolic syndrome alone contributed 8.4% of cases (Fig. 2; Table 3). Behavioural factors alone were not associated with any new cases; however, behavioural factors in conjunction with the metabolic syndrome were associated with 22.9% of cases. In conjunction with concurrent depression-anxiety, behavioural factors contributed an additional 1.4% of cases. The strata associated with the most incident diabetes cases was therefore the metabolic syndrome in conjunction with behavioural factors (PAF = 22.9%). The presence of either depression, anxiety or concurrent depression-anxiety conferred an additional 13.6% of cases to this risk factor combination (PAFs = 2.5%, 4.9%, and 6.2%, respectively). No diabetes cases in the overall sample were attributable to the presence of psychological risk factors alone (Table 3).

**Table 3:** Multivariate relative risks and population attributable fractions (PAF) for Type 2 diabetes onset in Norwegian adults associated with metabolic, behavioural and psychological risk factors (N = 36,161)

Behavioural^a^	MetSyn^b^	Depression	Anxiety	Concurrent dep–anx	# exposed	Exposure prevalence (%)	Diabetes cases	RR (95% CI)	PAF
0	0	0	0	0	7602	21.02	123	1.00	
0	0	1	0	0	803	2.22	10	0.93 (0.49, 1.76)	–
0	0	0	1	0	1461	4.04	13	0.56 (0.32, 0.98)	–
0	0	0	0	1	1319	3.65	25	1.31 (0.85, 2.00)	–
1	0	0	0	0	11,309	31.27	218	1.13 (0.91, 1.41)	–
1	0	1	0	0	1256	3.47	24	1.30 (0.85, 2.00)	–
1	0	0	1	0	2447	6.77	52	1.26 (0.91, 1.73)	–
1	0	0	0	1	2435	6.73	56	1.49 (1.09, 2.04)	1.4
0	1	0	0	0	1561	4.32	145	4.42 (3.49, 5.60)	8.4
0	1	1	0	0	135	0.37	15	6.87 (4.16, 11.35)	1.0
0	1	0	1	0	278	0.77	30	4.88 (3.17, 7.53)	1.8
0	1	0	0	1	255	0.71	22	5.46 (3.74, 7.97)	1.4
1	1	0	0	0	3493	9.66	376	5.26 (4.30, 6.42)	22.9
1	1	1	0	0	342	0.95	39	6.13 (4.36, 8.62)	2.5
1	1	0	1	0	766	2.12	81	5.29 (4.03, 6.93)	4.9
1	1	0	0	1	699	1.93	95	7.47 (5.79, 9.64)	6.2
		Total			36,161	100	1324		50.5

Adjusted for age and sex

^a^Physical inactivity and smoking factors

^b^Metabolic syndrome (as defined by the International Diabetes Federation [28])

**Fig. 3:**
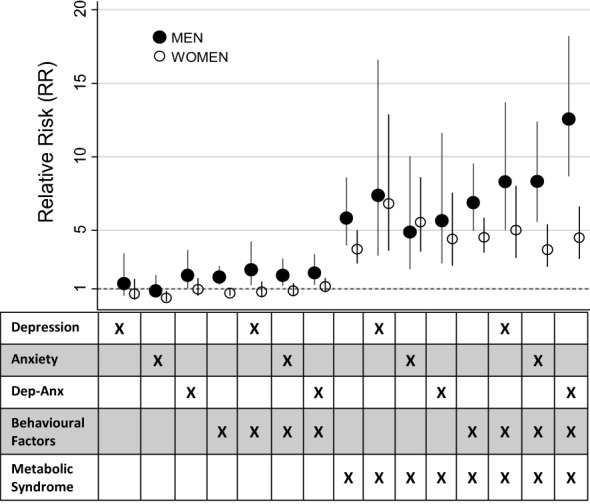
Relative Risk for Type 2 diabetes onset in Norwegian adults associated with multiple risk factors, by sex (N = 36,161)

The highest adjusted relative risk of incident diabetes was observed in individuals with all four risk factors (RR = 7.47 [95% CI 5.79, 9.64]), but this combination occurred in less than 2% of exposed individuals, resulting in a PAF of 6.2% overall. In men experiencing all four risk factors, the relative risk of diabetes was 12.57 (95% CI 8.67, 16.22) and the PAF was 8.4% (Additional file 1: Table S1; Fig. 2). These were substantially higher than the results observed in women (RR = 4.49 [95% CI 3.06, 6.60], PAF = 4.0%) (Additional file 2: Table S2; Fig. 3).

As illustrated in Fig. 3, the pattern of risk for diabetes incidence was more pronounced in men than in women. Behavioural factors alone conferred a substantial proportion of cases in men only (PAF = 8.9%). This proportion was even higher in the presence of depression (PAF = 10.9%). Conversely, a larger proportion of cases were attributable to the metabolic syndrome alone in women than men (PAFs = 9.8% and 6.7%, respectively). The total PAF for all risk factor combinations was 50.5% (women = 47.0%, men = 78.2%). In all instances, the Miettinen PAF estimates were more conservative than those calculated using Levin’s formula (Additional file 3: Table S3).

## Discussion

This study examined the population attributable fraction of Type 2 diabetes incidence associated with the metabolic syndrome, behavioural factors, and depression and anxiety in a cohort of Norwegian adults. Overall, these factors accounted for 50.5% of new diabetes cases, a proportion that was higher in men (78.2%) than women (47.0%). Symptoms of depression and anxiety contributed to diabetes incidence, but they did so only in conjunction with metabolic and behavioural risk factors. The group at highest risk for diabetes incidence was individuals exposed to both behavioural and metabolic factors (PAF = 22.9%); anxiety and depression contributed a further 13.6% of new cases to this combination. Men appeared to be particularly vulnerable to the interaction between metabolic, behavioural and psychological risk factors.

In the overall cohort, the PAFs associated with depression or anxiety alone were negligible, despite our multivariate analysis reflecting a relative risk of diabetes onset of roughly 1.40 for depression, which is in line with previous research [37]. However, as mentioned above, depression and anxiety in combination with behavioural and/or metabolic factors contributed to a substantial proportion of excess cases—a total of 19.2% across strata. For all strata containing depressive symptoms the PAF was 12.5%, considerably exceeding the 4.0% for depression alone estimated by Cosgrove et al. based on a risk estimate of 1.25 [15]. It is worth noting that the latter PAF was calculated using Levin’s formula and examined depression risk independently, both of which could overestimate the independent PAF associated with depression when compared to our calculations. Our findings suggest that the joint effect of these factors may be more relevant to diabetes onset than their independent effects, and underscore the potential influence of psychological factors on both metabolic and behavioural processes. While many relevant mediating relationships have been documented with respect to depression [4–6], symptoms of anxiety appeared to have a larger impact on these relationships in our study than depression (primarily due to the higher prevalence of anxiety in the population). Further studies in this area should examine interactions with traditional risk factors when attempting to model diabetes risk associated with depression or anxiety, as this may provide a truer depiction of psychological risk than is available in the literature to date.

With respect to the metabolic syndrome, a combined fraction of 49.1% of new diabetes cases was observed across all strata containing this factor. This is in line with previous research demonstrating a metabolic syndrome PAF of 30–52% for diabetes across multiple studies [19]. The independent effect of the metabolic syndrome alone conferred a higher proportion of cases in women (9.8%) than men (6.7%). This is somewhat unexpected given that men had higher frequencies of all metabolic risk factors at baseline, with the exception of central obesity, and may point to the increased tendency of men with the metabolic syndrome to experience behavioural and psychological comorbidities than women. This finding may be partly explained by the higher prevalence of central obesity in women (47.9% versus 36.9% in men), and is in line with previous research demonstrating that central obesity is a stronger predictor of metabolic syndrome status in women than men [38]. These findings suggest that women who do not have any concurrent behavioural or psychological risk may still be particularly vulnerable to the negative consequences of metabolic risk factors such as central obesity.

Conversely, smoking and physical inactivity alone did not independently confer diabetes risk in women in our study, yet accounted for 8.9% of incident cases in men. In conjunction with depression or anxiety, these factors were associated with another 15.0% of incident cases in men. One partial explanation for this sex-related difference may be the higher prevalence of former smoking reported by men, as well as the total number of packyears. The relationship between smoking and diabetes incidence is particularly pernicious in men, with research reflecting that men require double the length of time following smoking cessation to return to non-smoking diabetes risk levels (≥ 10 years, as opposed to ≥ 5 years in women) [39].

As illustrated in Fig. 3, the interaction of metabolic, behavioural, and psychological factors had a more profound impact on diabetes incidence in men than women. The relative risk was 12.5 times higher in men experiencing all four factors, accounting for 8.4% of new diabetes cases. By comparison, this risk was one third the size in women and accounted for only 4.0% of new cases. Previous research has demonstrated stronger effects of depression on glycemic control in men only [40], as well as the presence of elevated inflammation in men but not women with anxiety disorders [41]. Recent research also demonstrates higher mortality rates in men with Type 2 diabetes experiencing concurrent depression and anxiety than women [42]. It may be possible that men experiencing all four equivalent risk factors are more susceptible to their effects than women, or alternately that men reporting depression and anxiety at baseline tend to have a more severe metabolic or behavioural risk profile than women reporting these symptoms within the same strata. Future research should attempt to unpack these interactions within this high-risk group.

### Strengths and limitations

This study used a large population-based sample of Norwegian adults, and allowed for an appropriate follow-up time to assess diabetes incidence. We rigorously controlled for a range of clinical and sociodemographic covariates and exposures. We also used a formula for calculating population attributable risk that was valid in the presence of confounding and interaction, and provided an intuitive presentation of these results. Instead of focusing on the potential proportion of diabetes cases that could be reduced by eliminating depression or anxiety in the population overall, the current analysis allows an examination of strata in which targeted treatment of these symptoms might be more beneficial. For example, of our study population who experienced symptoms of depression and anxiety at baseline, only 13.6% of these individuals developed diabetes during follow-up, yet they accounted for 19.2% of new cases. Given these findings and the current diabetes epidemic, research to determine whether treating anxiety and depression in people exposed to other diabetes risk factors is effective in reducing the incidence rate may therefore prove to be useful [43].

However, we are unable to assume a direct causal link between depression and anxiety and diabetes incidence. As noted by Flegal et al. direct causal assumptions are not necessarily warranted when using PAFs [44]—as a result of mediational processes, different methods of intervening on a modifiable factor such as obesity may lead to different counterfactual outcomes, even if they achieve identical reductions in weight [45]. We were also unable to calculate *partial* or *summary attributable risks* as not all relative risks were greater than 1, and were therefore unable to report the summed contributions of each individual risk factor (i.e., ‘column totals’). This highlighted the importance of the interaction between psychological factors and metabolic and behavioural factors in eliciting effects on diabetes onset.

In addition, as risk factors were measured simultaneously at baseline, it was not possible to assess mediating relationships or to differentiate between distal and proximal mechanisms of risk underlying these associations. Although the diabetes risk related to behavioural factors was relatively low in this study, these factors tend to confer disease risk through their subsequent effects on metabolic processes. For example, physical inactivity or smoking in individuals prior to baseline may have precipitated changes in metabolic health which persisted even if their behavioural risk status had changed by the time of assessment. It is therefore likely that we have underestimated the true impact of behavioural factors in the present study. Previous research on non-participation in the HUNT 3 study has also demonstrated that both mental health and diabetes status are linked to study non-participation [46], and it is therefore possible that this study underestimated the true incidence rate of diabetes in the Norwegian population. As with all observational studies, our results are also vulnerable to the presence of unmeasured confounding.

It is worth noting that PAFs are highly influenced by the population prevalence of the risk factor of interest. In the aforementioned study of sugar sweetened beverages, the authors reported that while the PAF for diabetes cases in the USA was 8.7%, it was only 3.6% in the UK. These values may therefore vary in countries outside of Norway, where the prevalence of diabetes risk factors differs. Future research should quantify these effects within each target population of interest prior to using them to inform local health strategies.

In addition, it is worth noting that not all individuals exposed to the same complement of risk factors will display the same diabetes phenotype. Many genetic factors, gene–gene and gene-environment interactions can affect the onset and type of diabetes mellitus. We did not have access to genetic data for this study, and the addition of this data could prompt an interesting extension of the current research.

## Conclusions

Overall, the presence of the metabolic syndrome in conjunction with behavioural factors was the largest contributor to diabetes incidence in the Norwegian population. In addition, symptoms of depression and anxiety increased the percentage of excess diabetes cases attributable to common behavioural and metabolic factors, particularly in men. This study highlights the importance of risk factor clustering in diabetes onset, and is the first that we know of to quantify the excess fraction of incident diabetes associated with psychological risk factor interactions.

## Additional files

**Additional file 1: Table S1.** Multivariate relative risks and PAFs for Type 2 diabetes onset in Norwegian adults associated with metabolic, behavioural and psychological risk factors, women only (N=20,014)*.

**Additional file 2: Table S2.** Multivariate relative risks and PAFs for Type 2 diabetes onset in Norwegian adults associated with metabolic, behavioural and psychological risk factors, men only (N=16,147)*.

**Additional file 3: Table S3.** Comparison of Miettinen and Levin’s formulas for calculating population attributable fractions (PAFs).

## Abbreviations

AUC: area under the curve
CI: confidence interval
HDL: high-density lipoprotein
PAF: population attributable fraction
RR: relative risk
