# Supplementary material for: Population attributable fractions for Type 2 diabetes: an examination of multiple risk factors including symptoms of depression and anxiety
Source: Diabetol Metab Syndr. 2018 Nov 22;10:84. doi: 10.1186/s13098-018-0387-5 (PMC6251110; doi:10.1186/s13098-018-0387-5)
Supplement: Supplementary file 1 — Additional file 1: Table S1. Multivariate relative risks and PAFs for Type 2 diabetes onset in Norwegian adults associated with metabolic, behavioural and psychological risk factors, women only (N=20,014)*. [file 13098_2018_387_MOESM1_ESM.docx]

Table S1. Multivariate relative risks and PAFs for Type 2 diabetes onset in Norwegian adults associated with metabolic, behavioural and psychological risk factors, women only (N=20,014)*

| Behavioural^†^ | MetSyn^‡^ | Depression | Anxiety | Concurrent Dep-anx | # exposed | Exposure prevalence | Diabetes cases | RR (95% CI) | PAF |
| --- | --- | --- | --- | --- | --- | --- | --- | --- | --- |
| 0 | 0 | 0 | 0 | 0 | 4,202 | 21.00% | 80 | 0.0 |  |
|  | | | | | | | | | |
| 0 | 0 | 1 | 0 | 0 | 481 | 2.40% | 5 | 0.68 (0.28, 1.68) | - |
| 0 | 0 | 0 | 1 | 0 | 811 | 4.05% | 6 | 0.39 (0.17, 0.88) | - |
| 0 | 0 | 0 | 0 | 1 | 798 | 3.99% | 13 | 0.96 (0.54, 1.72) | - |
|  | | | | | | | | | |
| 1 | 0 | 0 | 0 | 0 | 6,185 | 30.90% | 86 | 0.73 (0.54, 0.98) | - |
| 1 | 0 | 1 | 0 | 0 | 824 | 4.12% | 11 | 0.81 (0.43, 1.51) | - |
| 1 | 0 | 0 | 1 | 0 | 1,342 | 6.71% | 23 | 0.88 (0.56, 1.40) | - |
| 1 | 0 | 0 | 0 | 1 | 1,469 | 7.34% | 30 | 1.16 (0.76, 1.76) | - |
|  | | | | | | | | | |
| 0 | 1 | 0 | 0 | 0 | 905 | 4.52% | 88 | **3.70 (2.74, 4.99)** | 9.8 |
| 0 | 1 | 1 | 0 | 0 | 70 | 0.35% | 9 | **6.80 (3.60, 12.89)** | 1.2 |
| 0 | 1 | 0 | 1 | 0 | 162 | 0.81% | 22 | **4.40 (2.57, 7.56)** | 2.6 |
| 0 | 1 | 0 | 0 | 1 | 150 | 0.75% | 14 | **5.53 (3.54, 8.62)** | 1.8 |
|  | | | | | | | | | |
| 1 | 1 | 0 | 0 | 0 | 1,680 | 8.39% | 180 | **4.51 (3.48, 5.84)** | 21.4 |
| 1 | 1 | 1 | 0 | 0 | 185 | 0.92% | 19 | **5.00 (3.11, 8.02)** | 2.3 |
| 1 | 1 | 0 | 1 | 0 | 394 | 1.97% | 35 | **3.67 (2.50, 5.39)** | 3.9 |
| 1 | 1 | 0 | 0 | 1 | 356 | 1.78% | 34 | **4.49 (3.06, 6.60)** | 4.0 |
| TOTAL | | | | | 20,014 | 100% | 655 | **-** | 47.0 |

* Adjusted for age and sex
^†^ Physical inactivity and smoking factors
^‡^ Metabolic syndrome (as defined by the International Diabetes Federation (28))
