# Supplementary material for: Population attributable fractions for Type 2 diabetes: an examination of multiple risk factors including symptoms of depression and anxiety
Source: Diabetol Metab Syndr. 2018 Nov 22;10:84. doi: 10.1186/s13098-018-0387-5 (PMC6251110; doi:10.1186/s13098-018-0387-5)
Supplement: Supplementary file 2 — Additional file 2: Table S2. Multivariate relative risks and PAFs for Type 2 diabetes onset in Norwegian adults associated with metabolic, behavioural and psychological risk factors, men only (N=16,147)*. [file 13098_2018_387_MOESM2_ESM.docx]

Table S2. Multivariate relative risks and PAFs for Type 2 diabetes onset in Norwegian adults associated with metabolic, behavioural and psychological risk factors, men only (N=16,147)*

| Behavioural^†^ | MetSyn^‡^ | Depression | Anxiety | Comorbid Dep-anx | # exposed | Exposure prevalence | Diabetes cases | RR (95% CI) | PAR |
| --- | --- | --- | --- | --- | --- | --- | --- | --- | --- |
| 0 | 0 | 0 | 0 | 0 | 3,400 | 21.03% | 43 | 0.0 | - |
|  | | | | | | | | | |
| 0 | 0 | 1 | 0 | 0 | 322 | 1.99% | 5 | 1.37 (0.55, 3.43) | 2.0 |
| 0 | 0 | 0 | 1 | 0 | 650 | 4.03% | 7 | 0.88 (0.40, 1.95) | - |
| 0 | 0 | 0 | 0 | 1 | 521 | 3.23% | 12 | **1.94 (1.03, 3.65)** | 0.9 |
|  | | | | | | | | | |
| 1 | 0 | 0 | 0 | 0 | 5,124 | 31.73% | 132 | **1.82 (1.30, 2.56)** | 8.9 |
| 1 | 0 | 1 | 0 | 0 | 432 | 2.68% | 13 | **2.30 (1.25, 4.23)** | 10.9 |
| 1 | 0 | 0 | 1 | 0 | 1,105 | 6.84% | 29 | **1.93 (1.21, 3.07)** | 2.1 |
| 1 | 0 | 0 | 0 | 1 | 966 | 5.98% | 26 | **2.08 (1.29, 3.37)** | 2.0 |
|  | | | | | | | | | |
| 0 | 1 | 0 | 0 | 0 | 656 | 4.06% | 57 | **5.83 (3.96, 8.58)** | 6.7 |
| 0 | 1 | 1 | 0 | 0 | 65 | 0.40% | 6 | **7.37 (3.28, 16.59)** | 0.8 |
| 0 | 1 | 0 | 1 | 0 | 116 | 0.72% | 8 | **4.85 (2.35, 10.01)** | 0.9 |
| 0 | 1 | 0 | 0 | 1 | 105 | 0.65% | 8 | **5.64 (2.74, 11.63)** | 1.0 |
|  | | | | | | | | | |
| 1 | 1 | 0 | 0 | 0 | 1,813 | 11.23% | 196 | **6.87 (4.96, 9.52)** | 24.9 |
| 1 | 1 | 1 | 0 | 0 | 157 | 0.97% | 20 | **8.29 (5.02, 13.70)** | 2.6 |
| 1 | 1 | 0 | 1 | 0 | 372 | 2.30% | 46 | **8.30 (5.56, 12.39)** | 6.1 |
| 1 | 1 | 0 | 0 | 1 | 343 | 2.12% | 61 | **12.57 (8.67, 16.22)** | 8.4 |
| TOTAL | | | | | 16,147 | 100% | 669 | **-** | 78.2 |

* Adjusted for age and sex
^†^ Physical inactivity and smoking factors
^‡^ Metabolic syndrome (as defined by the International Diabetes Federation (28))
