# Supplementary material for: Population attributable fractions for Type 2 diabetes: an examination of multiple risk factors including symptoms of depression and anxiety
Source: Diabetol Metab Syndr. 2018 Nov 22;10:84. doi: 10.1186/s13098-018-0387-5 (PMC6251110; doi:10.1186/s13098-018-0387-5)
Supplement: Supplementary file 3 — Additional file 3: Table S3. Comparison of Miettinen and Levin’s formulas for calculating population attributable fractions (PAFs). [file 13098_2018_387_MOESM3_ESM.docx]

Table S3. Comparison of Miettinen and Levin’s formulas for calculating population attributable fractions (PAFs)

| Behavioural | MetSyn | Depression | Anxiety | Concurrent Dep-anx | ALL | | WOMEN | | MEN | |
| --- | --- | --- | --- | --- | --- | --- | --- | --- | --- | --- |
|  | | | | | Miettinen | Levin | Miettinen | Levin | Miettinen | Levin |
| 0 | 0 | 0 | 0 | 0 |  |  |  |  | - |  |
|  | | | | | | | | | | |
| 0 | 0 | 1 | 0 | 0 | - | - | - | - | 2.0 | 0.7 |
| 0 | 0 | 0 | 1 | 0 | - | - | - | - | - | - |
| 0 | 0 | 0 | 0 | 1 | - | - | - | - | 0.9 | 2.9 |
|  | | | | | | | | | | |
| 1 | 0 | 0 | 0 | 0 | - | - | - | - | 8.9 | 20.6 |
| 1 | 0 | 1 | 0 | 0 | - | - | - | - | 10.9 | 3.4 |
| 1 | 0 | 0 | 1 | 0 | - | - | - | - | 2.1 | 6.0 |
| 1 | 0 | 0 | 0 | 1 | 1.4 | 3.2 | - | 1.1 | 2.0 | 6.1 |
|  | | | | | | | | | | |
| 0 | 1 | 0 | 0 | 0 | 8.4 | 12.9 | 9.8 | 10.9 | 6.7 | 16.4 |
| 0 | 1 | 1 | 0 | 0 | 1.0 | 2.1 | 1.2 | 2.0 | 0.8 | 2.5 |
| 0 | 1 | 0 | 1 | 0 | 1.8 | 2.9 | 2.6 | 2.7 | 0.9 | 2.7 |
| 0 | 1 | 0 | 0 | 1 | 1.4 | 3.1 | 1.8 | 3.3 | 1.0 | 2.9 |
|  | | | | | | | | | | |
| 1 | 1 | 0 | 0 | 0 | 22.9 | 29.1 | 21.4 | 22.7 | 24.91 | 39.7 |
| 1 | 1 | 1 | 0 | 0 | 2.5 | 4.7 | 2.3 | 3.5 | 2.6 | 6.6 |
| 1 | 1 | 0 | 1 | 0 | 4.9 | 8.3 | 3.9 | 5.0 | 6.1 | 14.4 |
| 1 | 1 | 0 | 0 | 1 | 6.2 | 11.1 | 4.0 | 5.8 | 8.4 | 19.7 |
|  |  | **TOTAL** |  |  | **50.5** | **77.4** | **47.0** | **55.9** | **78.2** | **144.6** |
